# Dataset of high-speed camera measurements from impact-tested reinforced concrete beams

**DOI:** 10.1016/j.dib.2026.112487

**Published:** 2026-01-21

**Authors:** Viktor Peterson

**Affiliations:** Division of Concrete Structures, KTH Royal Institute of Technology, 10044 Stockholm, Sweden

**Keywords:** Shear failure, Digital image correlation, Photron Fastcam APX-RS, HBM QuantumX MX440B, B&K Type 4375

## Abstract

Impact-loaded reinforced concrete beams often fail in shear. This becomes relevant for shelter design against ballistics or fragment impact, for instance. An experimental campaign was conducted to study the different types of shear failure and governing parameters. Eighteen reinforced concrete beams were tested by a 70 kg steel striker dropped from a 2.4 m height. The beams were loaded at different positions from the support with different amounts of transverse reinforcement. The beams were of reduced scale with a length of 0.80 m and a square 0.15 m × 0.15 m cross-section. The drop weight tests were monitored with shock accelerometers on the striker and beam centre, load cells under the supports measuring reaction forces, and a high-speed camera (HSC). High-speed camera measurements were recorded orthogonal to the surface with the aim of performing high-quality digital image correlation (DIC) analyses. The beams and striker were painted with a speckled pattern prior to testing for the DIC analyses. Camera recordings were conducted with a 1024 × 512 px resolution and 6 kHz sampling, resulting in a time resolution of about 0.17 ms. Accelerometer and load cell measurements were sampled at 19.2 kHz. The accelerometer on the striker was used to approximate the impact force, and beam acceleration can be used to synchronize the camera and DAQ recordings. The data may be used to calibrate finite element models, study the impact response of beams, or develop new mechanical models.

Specifications Table

**Table:** 

Subject	Engineering & Materials science
Specific subject area	Impact-loaded beams monitored with high-speed cameras for digital image correlation analyses
Type of data	Raw high-speed camera images Raw load cell sensor data Raw accelerometer sensor data
Data collection	High-speed camera measurements were collected by a Photron Fastcam APX-RS camera. Recordings were conducted with a 1024 × 512 px resolution with a 6 kHz sampling. The beams and striker were painted with a high-contrast speckled pattern prior to testing for the subsequent DIC analyses. Shock accelerometer (B&K Type 4375) and load cell (Load indicator Kraftgivare AB) data were recorded using a DAQ (HBM QuantumX MX440B) sampled at 19.2 kHz.
Data source location	• KTH Royal Institute of Technology • Stockholm • Sweden
Data accessibility	Repository name: Mendeley Data Data identification number: 10.17632/kn28g6dbj5.3 Direct URL to data: https://data.mendeley.com/datasets/kn28g6dbj5/3
Related research article	V. Peterson, J. Magnusson, M. Hallgren, A. Ansell. Shear-type failure of deep, short and slender impact-loaded reinforced concrete beams. International Journal of Impact Engineering, 2026, 208, 105539, 10.1016/j.ijimpeng.2025.105539.

## 1 Value of the Data

• The data may be used to study the dynamic shear-carrying mechanisms and general impact response of reinforced concrete beams with and without transverse reinforcement. Different types of shear failures were provoked.
• The data may be used to validate and develop mechanical models for impact-loaded beams, including global response models (such as single-degree-of-freedom models) and shear capacity models.
• The data may be used to validate and develop numerical models. Such calibrated models could be used to extend the test series, or for new types of tests.
• The data may be used to validate new methods for digital image correlation analyses of dynamic systems. The results of DIC analyses from camera measurements may be compared to accelerometer signals.

The dataset is relevant to multiple research communities, including (i) structural impact and blast engineering, (ii) reinforced concrete shear and structural dynamics, (iii) computational mechanics and finite-element modelling of RC under high strain-rates, and (iv) experimental mechanics and full-field measurement methods (e.g., digital image correlation). It can support researchers developing and validating shear-capacity formulations, global response models (e.g., single-degree-of-freedom approaches), and high-fidelity numerical models (e.g., detailed FE and smeared-crack/constitutive models), as well as researchers benchmarking DIC workflows for dynamic tests against sensor-based measurements. Potential users also include guideline and research organisations active in structural concrete and protective design (e.g., fib, RILEM, ACI technical communities).

The dataset is also useful for professional stakeholders involved in designing, assessing, and managing structures exposed to accidental or intentional impact actions. This includes structural engineering consultancies working with protective design, owners/operators of transport infrastructure (e.g., bridges, road and rail authorities), and organisations responsible for safety and resilience of critical infrastructure (e.g., public works agencies and asset managers). These stakeholders can use the data to improve understanding of impact-induced shear failure modes in RC beams (with and without transverse reinforcement), to calibrate or sanity-check simplified assessment tools, and to verify numerical modelling strategies used in design studies, retrofit evaluations, and fragility/risk-informed assessments.

## 2 Background

These data were collected to study shear-type failures of impact-loaded beams as part of a research project on shear failures of reinforced concrete structural elements under blast and impact. For this aim, DIC analyses of motion and strain were conducted from high-speed camera measurements. The data were synced to signals from a DAQ system with shock accelerometer sensors and load cells. This allowed the study of the dynamic capacity, dynamic response and shear-carrying mechanisms. A future aim is to develop calibrated numerical models that could be used for extended studies of the dynamic response of impact-loaded reinforced concrete beams with and without transverse reinforcement.

There is a need for high-quality data for validation of numerical models. Calibrated and validated numerical models may be used instead of experimental investigation. This dataset allows for accessibility of impact-loaded beam research for the general research community where expensive sensors may not be accessible. There are already examples where the dataset has been used for model calibration and extended studies [1,2].

## 3 Data Description

The naming convention for the 18 specimens is given in Table 1. The data are divided into two main folders, with one subfolder for each specimen:

• DAQ System Measurements
  ○ D-04d-NoS-1
  ○ D-04d-NoS-2
  ○ …
• HSC Measurements
  ○ D-04d-NoS-1
  ○ D-04d-NoS-2
  ○ …

**Table 1:** The names of the specimens are defined as follows: D-YY-ZZZ-W. Here, D represents dynamic loading, YY represents the short shear span length expressed in terms of the effective depth (0.4d, 1d or 2d), ZZZ represents the stirrup spacing (NoS is no stirrups, S90 for 90 mm spacing, and S45 for 45 mm spacing).

Specimen	Shear span length	Stirrup spacing (mm)	Notes
D-04d-NoS-1	04d	-	
D-04d-NoS-2	04d	-	
D-04d-S90-1	04d	90	
D-04d-S90-2	04d	90	No DAQ data
D-04d-S45-1	04d	45	
D-04d-S45-2	04d	45	No DAQ data
D-1d-NoS-1	1d	-	
D-1d-NoS-2	1d	-	
D-1d-S90-1	1d	90	
D-1d-S90-2	1d	90	
D-1d-S45-1	1d	45	
D-1d-S45-2	1d	45	
D-2d-NoS-1	2d	-	
D-2d-NoS-2	2d	-	
D-2d-S90-1	2d	90	
D-2d-S90-2	2d	90	
D-2d-S45-1	2d	45	
D-2d-S45-2	2d	45	

The DAQ System Measurements folder contains two files for each specimen (Example for specimen D_04d_NoS_1):

• D_04d_NoS_1_19200Hz.mat
  - Registered signals from the load cells and accelerometers.
  - Contains five channels with signals (CH1: Time (s), CH2: Load cell short span (kN), CH3: Load cell long span (kN), CH4: Striker accelerometer (m/s^2^), and CH5: Beam accelerometer (m/s^2^)).
• Load_data.m
  - Matlab code for loading the data.

The HSC Measurements folder contains two subfolders:

• Test: Contains monochrome .tif figures for dynamic response.
• Calibration: Contains one monochrome .tif figure with a calibrated 90 mm gauge that may be used to relate pixels with physical dimensions. The calibration photo is taken with the same camera settings as the dynamic test.

For specimens D-04d-S90-2 and D-04d-S45-2, only high-speed camera data are available; no DAQ files are provided (see Table 1). This was due to an amplifier malfunction during data acquisition. The high-speed camera images for these specimens remain fully usable for kinematic and DIC-based analyses.

The calibration photo is used to calibrate the physical pixel size between tests. The camera settings remained unchanged, and the beam was positioned at the same location. Users may apply the same calibration factor across tests but should verify using the calibration image due to small alignment differences.

The load cell and accelerometer signals in the provided .mat files are exported directly from the DAQ without any post-processing (no filtering, offset correction, resampling, or synchronization to the camera). The time vector is the DAQ sampling clock.

## 4 Experimental Design, Materials and Methods

**Fig. 1:**
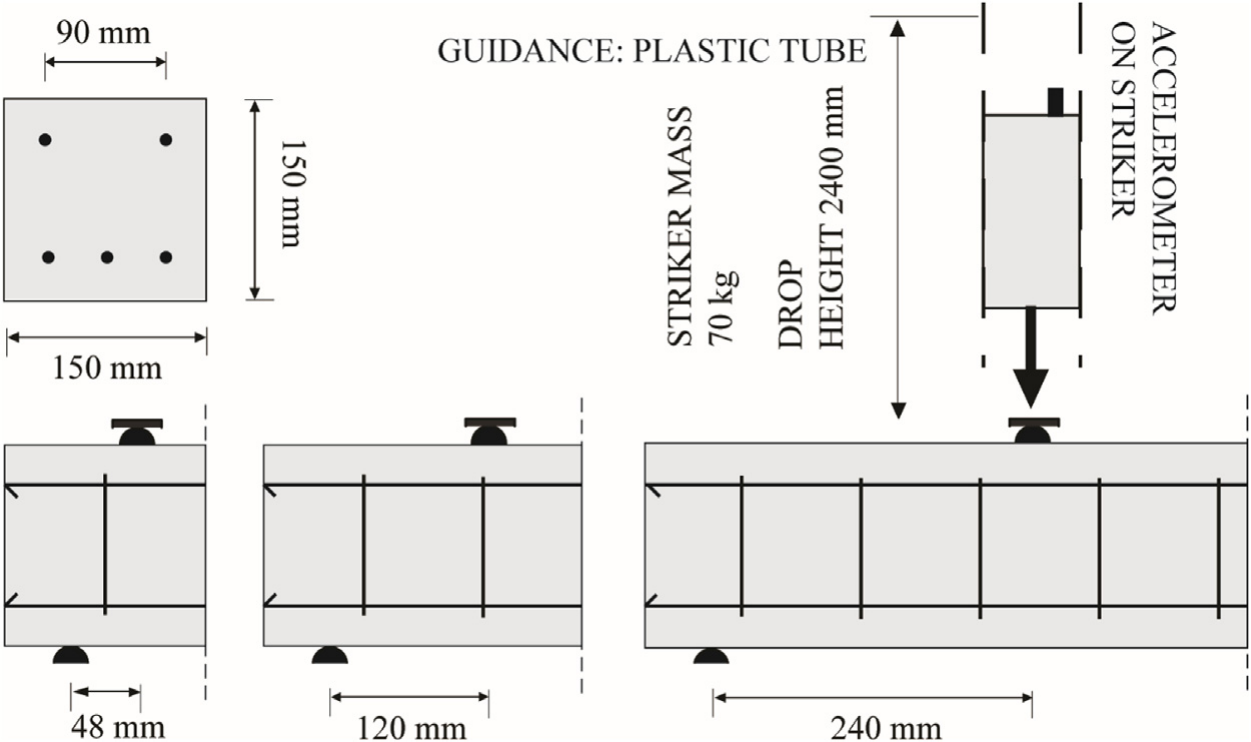
Test setup and geometry (redrawn from [3]).

Impact data were extracted from the instrumentation on the striker, beam and supports together with the high-speed camera. The tested beams are shown in Fig. 1. Beams were 0.80 m long with a 0.15 m × 0.15 m square cross-section. All beams contained the same longitudinal reinforcement, consisting of three 8 mm K500C reinforcement bars on the static tension side, and two 8 mm K500C reinforcement bars on the static compression side. The cover was 26 mm in all directions. Three stirrup configurations were tested: no stirrups, 6 mm stirrups at 90 mm spacing (75% of the effective depth), and 6 mm stirrups at 45 mm spacing (50% of the effective depth). The load was applied at three load positions from the left support: 48, 120, and 240 mm, corresponding to shear span-to-depth ratios of 0.4d, 1.0d, and 2.0d, respectively. This was done with the aim of provoking different shear-type failures.

The reinforcement was tested following the standards by the Swedish Standards Institute [4]. Three samples were tested per bar diameter. The mean yield and ultimate stresses for the 6 mm bars were 609 MPa and 701 MPa, respectively. For the 8 mm bars, the corresponding values were 511 MPa and 622 MPa. The uniaxial compression capacity was tested on 150 mm cubes, following Swedish Standards Institute [5]. From 16 samples, the mean compressive strength was 44 MPa with a standard deviation of 2 MPa. This corresponds approximately to concrete class C30/37.

The striker was equipped with a uniaxial accelerometer mounted near its top surface, and additional accelerometers were installed at the beam midspan on the top face. Load cells placed directly under each support recorded the time-varying support reactions. All analogue signals from accelerometers and load cells were routed through an HBM QuantumX MX440B amplifier and sampled at 19.2 kHz.

**Fig. 2:**
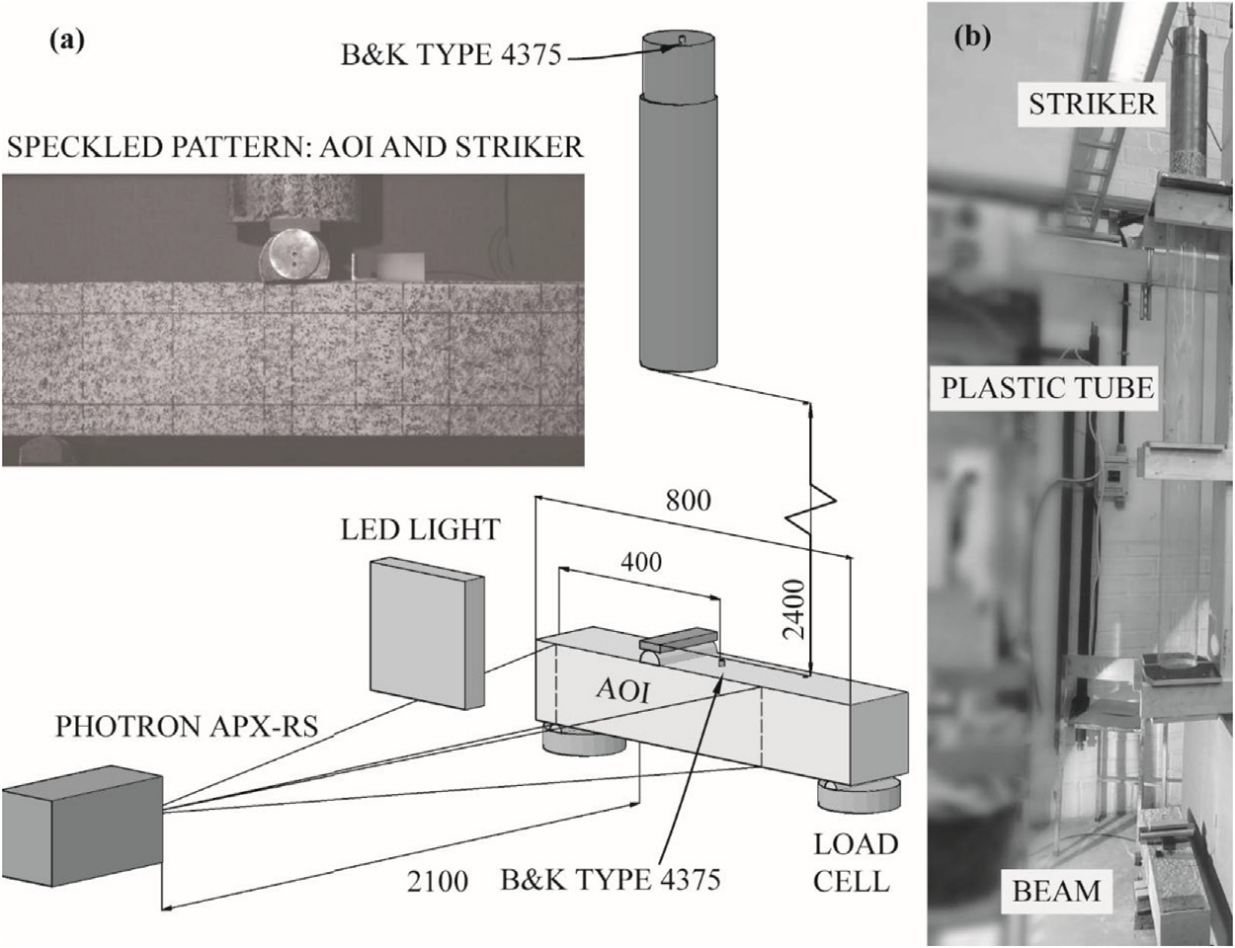
(a) Setup of the impact tests, and (b) photo of the testing rig (redrawn from [3]).

The high-speed optical recordings were acquired with the aim of enabling a later deformation and strain analysis by digital image correlation (DIC). The setup is shown in Fig. 2. All impact tests were recorded using a Photron APX-RS high-speed camera, positioned approximately orthogonal to one beam face and focused on the shear span where failure was expected. The camera was recording at 6 kHz with a resolution of 1024 × 512 px and a shutter speed of 1/5000 s. A 200 mm Nikon lens with f/4 aperture was used to obtain a sufficiently large field of view while maintaining spatial resolution. For each load position, an area of interest (AOI) was defined prior to testing: 350 mm (half the free span) for load positions 48 mm and 120 mm from the support, and 525 mm for the 240 mm load position. To provide a high-contrast texture suitable for later DIC tracking, all beams and the visible part of the striker were painted with a white base coat, followed by a random black speckle pattern applied using a natural sea sponge, following Johansson et al. [6]. High-power LED lamps were arranged to minimise shadows and glare and to ensure homogeneous illumination of the AOI. Prior to each impact, a 90 mm gauge block was placed in the beam plane at the impact region and recorded by the camera; these calibration images are stored together with the test recordings and allow a subsequent analyst to determine the physical pixel size and correct for any minor camera repositioning between tests.

It is easy to synchronise the data by comparing the DAQ signal of the beam or striker acceleration with the corresponding acceleration determined by DIC analysis from the images. The error may be minimised by maximising the cross-correlation between the signals. The potential error is in the magnitude of the order of the lower temporal resolution, i.e. 0.17 ms at 6000 fps.

## Limitations

Not applicable.

## Ethics Statement

The authors confirm that the ethical requirements have been read and are followed. The testing did not involve human subjects, animal experiments, or data from social media platforms.

## CRediT Author Statement

V. Peterson: Writing – original draft, Writing – review & editing, Visualization, Validation, Methodology, Investigation, Formal analysis, Data curation, Conceptualization.

## Data Availability

Mendeley DataHigh-speed camera dataset from impact tests on reinforced concrete beams (Original data)

## References

[1] E. Ceberg, and E. Holm, (2024). *Numerical Simulations of Impact-Loaded Reinforced Concrete Beams*. MSc Thesis, KTH Royal Institute of Technology, Div. Concrete Structures, Stockholm, Sweden, pp. 166.

[2] V. Peterson, E. Ceberg, E. Holm, E. Kolmodin, K. Kubiak, M. Hallgren, A. Ansell, (2025). *Effect of intense dynamic loads for reinforced concrete elements.* In: SILOS25. Gothenburg, Sweden, 11th to 13th June 2025, pp. 10.

[3] Peterson V., Magnusson J., Hallgren M., Ansell A. (2026). Shear-type failure of deep, short and slender impact-loaded reinforced concrete beams. Int. J. Impact Eng..

[4] Swedish Standards Institute (2019a). SS-EN ISO 6892-1:2019 Metallic materials – Tensile testing – Part 1: Method of test at room temperature. Stockholm, Sweden.

[5] Swedish Standards Institute (2019 b). SS-EN 12390-3:2019 Testing hardened concrete – Part 3: Compressive strength of test specimen. Stockholm, Sweden.

[6] Johansson M., Rempling R., Sanz-Diez de Ulzurrun Casals G., Zanuy C. (2019). IABSE Symposium Guimarães 2019 ‘Towards a Resilient Built Environment - Risk and Asset Management’.

